# There Are No Differences in Startle Conditioned Cervicomedullary Motor Evoked Potentials Across Isometric, Concentric, and Eccentric Muscle Actions at the Same Absolute Force Output

**DOI:** 10.1111/ejn.70205

**Published:** 2025-08-04

**Authors:** Eoin Haigney, Elliott Atkinson, Paul Ansdell, Rodrigo Vitorio, Kevin Thomas, Stuart Goodall, Glyn Howatson, Luca Angius, Dawson J. Kidgell, Padraig Spillane, Emma Squires, Justin W. Andrushko

**Affiliations:** ^1^ Department of Sport, Exercise and Rehabilitation, Faculty of Health and Life Sciences Northumbria University Newcastle upon Tyne Tyne and Wear UK; ^2^ Department of Psychology, Faculty of Health and Life Sciences Northumbria University Newcastle upon Tyne Tyne and Wear UK; ^3^ Physical Activity, Sport and Recreation Research Focus Area, Faculty of Health Sciences North‐West University Potchefstroom South Africa; ^4^ Water Research Group, School of Biological Sciences North‐West University Potchefstroom South Africa; ^5^ Kynisca Innovation Hub (KIH) Falls Church Virginia USA; ^6^ Monash Exercise Neuroplasticity Research Unit, School of Primary and Allied HealthCare Monash University Melbourne Australia

**Keywords:** cervicomedullary motor evoked potentials, human neuroscience, muscle contraction, neuromuscular physiology, peripheral nerve stimulation

## Abstract

Human movement involves a dynamic interplay of isometric, concentric, and eccentric muscle actions. There is a need to understand the contribution of the reticulospinal tract (RST) to human movement control during different muscle actions. This research aimed to determine the excitability of the RST during isometric, concentric, and eccentric muscle actions. Fourteen neurologically intact participants (age: 26 ± 7 years; sex: 3 female, 11 male; stature: 176 ± 8 cm; mass: 78.5 ± 10.9 kg) performed isometric, concentric, and eccentric muscle actions with the right *biceps brachii*. Participants performed a submaximal contraction at 25% of their isometric maximum voluntary contraction (MVC) during all muscle actions. Neurophysiological electrical stimulations to indirectly measure RST excitability consisted of conditioned (startling auditory stimulus of ≥ 110 dB) and unconditioned (no auditory stimulus) cervicomedullary motor evoked potentials (CMEPs). Larger conditioned CMEP responses compared with unconditioned CMEPs were observed for all muscle actions (*p* = 0.008). However, no differences in RST excitability, inferred from the difference between conditioned and unconditioned CMEP responses, were observed across the three muscle actions (*p* = 0.319). These results suggest that across isometric, concentric, and eccentric muscle actions, there are no differences in RST excitability while performing a submaximal contraction at 25% of their isometric MVC. It could therefore be inferred from this that RST input to motoneurons is not different between isometric, concentric, and eccentric muscle actions of the *biceps brachii* at a relatively low fixed absolute contraction intensity.

## Graphical abstract

In this work we demonstrate for the first time that reticulospinal tract (RST) excitability can be indirectly measured using conditioned cervicomedullary motor evoked potentials (CMEPs) during dynamic muscle actions. Although there were no differences in RST excitability across isometric, concentric and eccentric muscle actions during the same absolute force output, these findings support previous work that found the RST provides a generalized neural drive to support motor output.

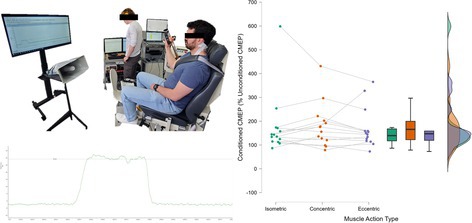

**Abbreviations**
CMEPs: cervicomedullary motor evoked potentials
CST: corticospinal tract
EHQ: Edinburgh handedness questionnaire
*M*_max_
: maximum compound muscle action potential
MVC: maximum voluntary contraction
M‐wave: compound muscle action potential
RM‐ANOVA: repeated measures analysis of variance
RMS: root mean square
RST: reticulospinal tract
sEMG: surface electromyography

## 1 Introduction

Motor function is critical for maintaining health and quality of life in older adults (Samuel et al. 2012), with muscle strength being a strong predictor of mortality and hospitalization in individuals over the age of 80 (Legrand et al. 2014). Importantly, the majority of daily tasks are performed at low sub‐maximal forces (Hortobagyi et al. 2001), and gaining a mechanistic understanding of the neuromuscular underpinnings that facilitate muscle function during low‐force muscle actions has wide‐reaching clinical and practical importance.

Previous research aiming to understand the neuromuscular underpinnings of strength has primarily focused on the role of the corticospinal tract (CST; the primary descending motor pathway) in muscle force production and adaptation, with equivocal outcomes (Ansdell et al. 2020; Kidgell et al. 2017; Pearcey et al. 2021; Siddique et al. 2020). The reticulospinal tract (RST) is a bilateral, descending pathway with the reticular nuclei originating from the pons and medulla in the brainstem. The RST receives inputs from several brain regions including the telencephalon, diencephalon, and the cerebellum (Brownstone and Chopek 2018), and synapses onto alpha‐ and gamma motoneurons via a mixture of direct monosynaptic and indirect polysynaptic connections (Riddle et al. 2009), with proximal muscles such as the elbow flexors predominantly receiving monosynaptic inputs (Riddle et al. 2009). Research has suggested the RST might play a larger role in strength output than previously thought (Atkinson et al. 2022; Glover and Baker 2020; Skarabot et al. 2021). In nonhuman primates, the RST has been shown to be the predominant site of neural adaptation to resistance training (Glover and Baker 2020), while in humans, similar observations have been observed with cross‐sectional data between chronically resistance trained and untrained participants (Akalu et al. 2024). Additionally, recent research has demonstrated brain activity in the pontine reticular nuclei (i.e., the origin of the RST) changes with force output (Danielson et al. 2024). Collectively, this emerging evidence suggests the RST plays a role in generating force output and adapts to facilitate increases in strength.

Thus far, examining the RST in humans has largely been limited to reaction time changes and isometric muscle actions, with only two studies examining the RST in dynamic upper limb movements (Altermatt et al. 2023; Maitland and Baker 2021). Yet none of the previous work has specifically examined how RST excitability differs between muscle action types. Human movement in naturalistic settings is a dynamic process involving not only isometric, but also concentric (i.e., where the muscle length shortens whilst producing force), and eccentric muscle actions (i.e., where the muscle produces force whilst lengthening). Previous research has identified greater CST excitability during concentric and isometric muscle actions compared with eccentric actions (Doguet et al. 2017; Sekiguchi et al. 2001, 2003), which aligns with observations of reduced spinal excitability during eccentric actions (Gruber et al. 2009). This suppression in excitability is thought to result from the actions of muscle spindle afferents, which inhibit cortical neurons and enhance presynaptic inhibition during muscle stretch (Skarabot et al. 2019a, 2019b). Additionally, studies using the decerebrate cat model have demonstrated that RST neurons are sensitive to manual stretch and direct stimulation of muscle afferents (Wolstencroft 1964). However, whether this sensitivity translates into modulation of RST excitability during different muscle actions in humans remains unclear.

Based on the aforementioned cat model, it is plausible that RST excitability is reduced to a greater extent during eccentric compared with concentric muscle actions, as eccentric actions are associated with greater activation of muscle spindle afferents. This heightened muscle spindle activity may lead to increased inhibitory feedback to alpha motoneurons, thereby diminishing excitatory drive from the RST (Del Valle and Thomas 2005). Understanding the role of the RST in different muscle actions may inform targeted interventions aimed at enhancing CST or RST adaptations. Such insights could be pivotal for developing strategies to optimize motor function and recovery in healthy aging, individuals with neurological impairments such as stroke, and during rehabilitation following injury.

Though it is expected RST excitability would be greatest in higher force muscle actions, ~97% of activities of daily living occur at contraction intensities lower than 30% maximum voluntary contraction (MVC) (Tikkanen et al. 2013). Therefore, the aim of this research was to test differences in RST excitability across different types of muscle actions (isometric, concentric, and eccentric) during low‐to‐moderate force in neurologically intact individuals. The hypothesis was that eccentric muscle actions would produce the lowest reticulospinal tract excitability with transmastoid stimulations via startle‐facilitated cervicomedullary motor evoked potentials (CMEPs) compared with isometric and concentric muscle actions.

## 2 Methodological Approach

### 2.1 Inclusion Criteria

For inclusion, participants were neurologically intact and between the ages of 18–45 years. Participants had no self‐reported hearing impairments and had not suffered any upper limb injuries in the previous year that caused sustained impairments or symptoms making elbow flexion contractions painful or unsafe. All participants passed standard screening for stimulation (Rossi et al. 2021). Additionally, hand dominance was assessed with the short form Edinburgh Handedness Questionnaire [EHQ; (Oldfield 1971; Veale 2014)] but participants were not restricted to a particular hand dominance. The EHQ is a 100 to −100 scale, where left hand dominance is any score in the range of −100 to −61, mixed hand dominance is represented by a score between −60 and 60, and right‐hand dominance is classified by scores between 61 and 100.

### 2.2 Participant Characteristics

Written informed consent was obtained from 17 participants (age: 26 ± 7 years; sex: 3 female, 14 male; stature: 177 ± 7 cm; mass: 78.2 ± 11.0 kg; handedness: 80.18 ± 47.81 EHQ) to participate in this study. However, two withdrew due to reported discomfort with the CMEP stimulation protocol, and 1 participant did not complete the protocol due to technological issues (hardware error, failed to detect signals from dynamometer). Therefore, a total of 14 (age: 26 ± 7 years; sex: 3 female, 11 male; stature: 177 ± 8 cm; mass: 78.5 ± 10.9 kg; handedness: 80.39 ± 52.51 EHQ) participants completed this study.

### 2.3 Ethical Approval

Ethical approval was obtained for this study from the Northumbria University Health and Life Sciences Research Ethics Committee (submission reference: #2024‐6411‐6888), and this study adhered to the ethical principles outlined in the Declaration of Helsinki (version 2024), with the exception of being registered in a database.

### 2.4 Design Overview

**FIGURE 1:**
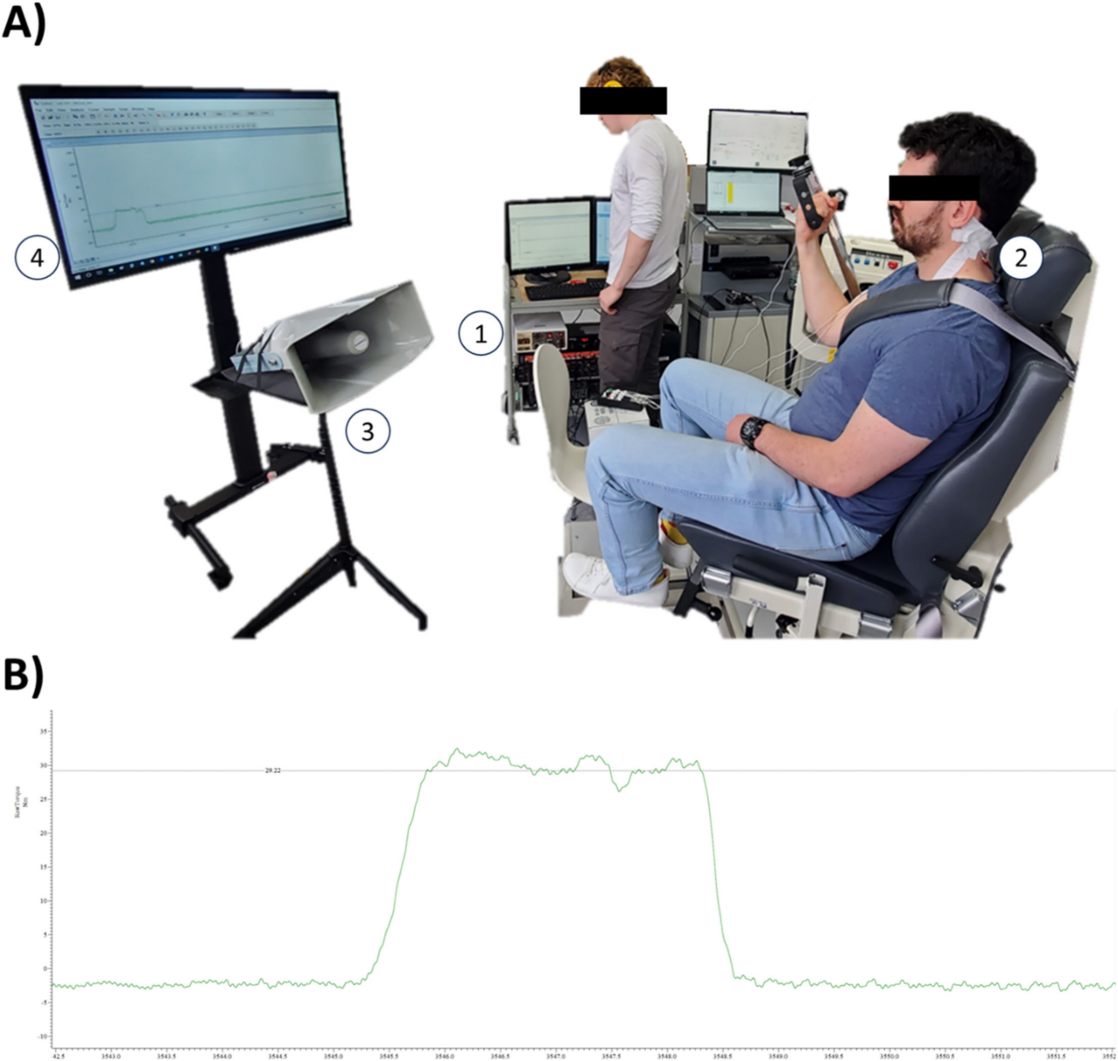
(A) Data collection setup. Numbers indicate primary components. 1: constant current stimulator; 2: cervicomedullary stimulation point on back of neck; 3: loudspeaker for auditory stimulation; 4: large monitor for target force presentation. The participant is sitting on a Biodex 3 isokinetic dynamometer, and (B) a visual representation of the task, where participants were instructed to contract to a visual horizontal target line.

This study implemented a within‐subject randomized design, where participants attended a single laboratory session and performed three types of muscle actions with their right arm: isometric, concentric, eccentric. Prior to data collection for each muscle action type, participants received standardized verbal instructions on task execution. They then completed multiple practice trials (typically 3–5 repetitions), with approximately 1 min of rest between attempts, to ensure accurate performance. Additional practice was provided if necessary; however, no participant required more than eight repetitions to meet the performance criteria. Data collection commenced only once participants could consistently perform the task while maintaining the target force throughout the entire range of motion. Figure 1 illustrates the laboratory setup used during testing sessions.

To minimize fatigue and avoid muscle damage, the order of muscle actions was partially randomized, starting with either concentric or isometric muscle actions, with eccentric muscle actions always being performed last (Proske and Morgan 2001). Each muscle action was performed under three stimulus conditions:

1. Conditioned CMEP: Loud startle auditory stimulus (≥ 110 dB) preceding transmastoid stimulation by 80 ms.
2. Unconditioned CMEP: Transmastoid stimulation without sound.
3. No sound + no stimulation: control condition.

**FIGURE 2:**
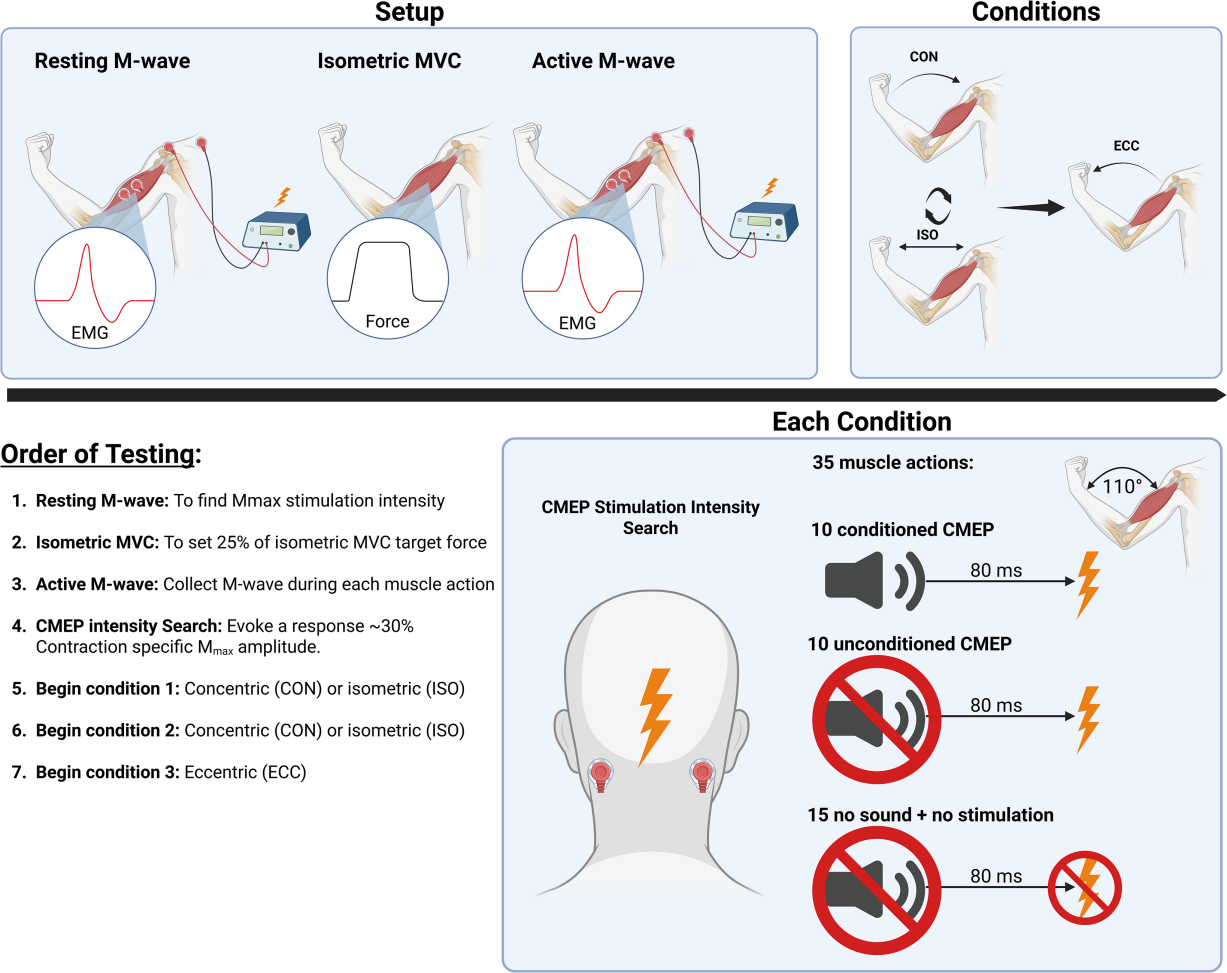
Study order of testing. Created in https://BioRender.com.

Each condition was repeated on 35 occasions during each muscle action type, with trials quasi‐randomized to avoid consecutive repetitions of the same stimulus type. The study procedures are outlined in detail in the order they took place (Figure 2).

#### 2.4.1 Surface Electromyography (sEMG)

For the *biceps brachii*, silver/silver chloride 28 mm × 20 mm (length × width) solid gel recording electrodes were used (Ambu Neuroline 710, Ballerup, Denmark). For the *biceps brachii*, electrodes were positioned along the line extending from the medial acromion to the fossa cubit, specifically at one‐third of the distance from the cubital fossa, in accordance with SENIAM guidelines (Hermens et al. 2000). As per SENIAM guidelines, electrodes were placed with an inter‐electrode distance of 2 cm in a bipolar arrangement, and a common ground reference electrode was placed on the head of the ulna (i.e., bony aspect at the wrist). Further sEMG details are provided in the signal acquisition section.

#### 2.4.2 Resting Muscle Response (M‐Wave)

To begin the session, involuntary maximum evoked elbow flexion muscle responses (*M*_max_) were assessed via electrical nerve stimulation of the musculocutaneous nerve over Erb's point (Lockyer et al. 2019). Resting M‐waves were collected to determine the stimulation intensity needed to evoke an *M*_max_. A DS7AH constant current stimulator (square wave 200 μs pulse width; Digitimer Ltd., Welwyn Garden City, Hertfordshire, UK) with 3.2 cm diameter PALS Neurostimulation Electrodes (Axelgaard Manufacturing Co., LTD. Fallbrook, CA, USA) was used to deliver a supramaximal current (mA) required to reach *M*_max_ plus 10%. The cathode electrode was placed on the skin of the supraclavicular fossa, and the anode electrode was placed on the acromion process. M‐waves were assessed at rest with a 110° elbow flexion joint angle. To begin, a low level of current was used to determine the electrode placement and then gradually increased until a plateau in the *biceps brachii* M‐wave was observed.

#### 2.4.3 Maximum Voluntary Contractions (MVC)

Following resting M‐wave stimulations, participants performed three isometric MVCs. Each MVC was 3 s in duration, with 2 min rest between attempts. The peak MVC was used to set a 25% isometric MVC target for performing the tasks for all three muscle action types. This ensured that the absolute force production was matched between muscle actions.

#### 2.4.4 Contraction Specific Muscle Response (Dynamic M‐Waves)

Using the stimulation intensity previously determined to evoke the resting *M*_max_ + 10%, three dynamic M‐wave stimulations were delivered at 110° of elbow flexion during isometric, concentric, and eccentric muscle actions at 25% of isometric MVC. The dynamic muscle actions were assessed during a constant velocity of 15°/second, and the stimulations were automatically triggered within Spike2 software (version 10.19) by setting a positional threshold trigger (i.e., the voltage from the Biodex dynamometer) corresponding to the participant's joint angle of 110° of elbow flexion. Dynamic M‐waves were recorded to normalize the contraction specific CMEP responses with their respective dynamic *M*_max_. While previous research suggests there are no significant differences in *M*_max_ amplitude between contraction types (Tallent et al. 2019), we collected *M*_max_ during each muscle action type to ensure CMEPs were normalized to the most context‐relevant evoked response. Although we acknowledge that it may not have been strictly necessary based on existing evidence (Tallent et al. 2019).

#### 2.4.5 Muscle Actions

Isometric, concentric, and eccentric muscle actions were performed at 25% of isometric MVC. The order of the first two muscle action types was randomized between isometric and concentric, with eccentric muscle actions always occurring as the third muscle action to avoid the impact of fatigue or muscle damage (Proske and Morgan 2001). A total of 35 muscle actions were performed for each muscle action type, with a minimum of 30 s rest between attempts. Isometric muscle actions were performed at 110° of elbow flexion, each three seconds in duration. Concentric and eccentric muscle actions were performed through 40° of motion, with a 20° range of motion on both sides of the 110° elbow flexion point (90°–130° range of motion) at a constant velocity of 15°/second. Of the 35 muscle actions, 15 no sound + no stimulation stimuli were randomly interspersed to avoid anticipation of the electrical and sound stimuli. A visual horizontal target representing 25% of isometric MVC was present on a TV screen (Figure 1B) and participants were instructed to reach this target force and maintain it throughout the entire range of motion during concentric and eccentric muscle actions.

#### 2.4.6 Auditory Stimuli

For each muscle action, participants completed three different stimulus conditions across 35 trials. Ten trials involved muscle actions performed without auditory stimuli but included transmastoid stimulation (unconditioned CMEP). Ten trials involved a loud auditory “startle” sound at ≥ 110 dB (500 Hz sine wave, 50‐ms duration) followed by transmastoid stimulation [conditioned CMEP; (Sangari and Perez 2020; Germann and Baker 2021)]. The remaining 15 trials involved muscle actions performed without auditory stimuli or transmastoid stimulation (no sound + no stimulation). The sequence of conditions was quasi‐randomized to prevent more than two consecutive conditioned or unconditioned trials. A loudspeaker (Adastra 40 W, 100‐V Horn PA Speaker) positioned 1 m directly in front of the participant delivered the auditory stimuli. During concentric and eccentric muscle actions, auditory stimuli were synchronized with joint angles reaching 110° of elbow flexion using Spike2 software and position signals from the Biodex system. For isometric muscle actions, the auditory stimuli were manually triggered once participants reached the target force of 25% MVC. The joint angle of 110° of elbow flexion was chosen based on previous findings by Chang, Su (Chang et al. 1999) who demonstrated that this position corresponds to the optimal muscle length for the biceps brachii, minimizing variability via a reduction in reflex input.

#### 2.4.7 Reticulospinal Tract Excitability

Transmastoid stimulations were delivered over the mastoid process 80 ms after the onset of each auditory stimulus condition (unconditioned CMEP, conditioned CMEP) to evoke CMEPs (Furubayashi et al. 2000). Stimulation was performed using a DS7AH constant current stimulator (200 μs square wave pulse width) with 3.2‐cm diameter PALS Neurostimulation Electrodes. Current direction was oriented towards the active limb, with the anode and cathode positioned contralateral and ipsilateral, respectively, to the contracting biceps brachii. The stimulation intensity was calibrated during a 25% MVC specific to each muscle action type and set to evoke CMEP peak‐to‐peak amplitudes approximately 30% (range: 25%–35%) of the contraction specific *M*_max_ amplitude (Weavil et al. 2018) prior to beginning the condition specific data collection. Differences in CMEP peak‐to‐peak amplitude between the conditioned and unconditioned stimuli for each muscle action type were analyzed to assess RST excitability.

The 80‐ms interstimulus interval was selected based on findings by Furubayashi et al. (2000), which demonstrated this interval optimizes response amplitudes during electrical stimulation. This methodology of conditioning CMEPs with auditory stimuli (> 110 dB) 80 ms prior to transmastoid stimulation to assess RST excitability aligns with established protocols (Sangari and Perez 2020; Germann and Baker 2021).

#### 2.4.8 Outcome Measure

The primary outcome measure was the difference in *biceps brachii* CMEP peak‐to‐peak amplitude between the conditioned CMEP and the unconditioned CMEP for each muscle action type (isometric, concentric, eccentric).

## 3 Signal Acquisition

### 3.1 sEMG

Surface EMG was collected with a Cambridge Electronic Design (CED) 1902 isolated preamplifier system (Cambridge, UK), with a gain of 1000, a common mode rejection ratio > 100 dB, and 10–500‐Hz fourth‐order Butterworth bandpass filtered.

### 3.2 Torque and Position

The torque signal from the Biodex was fed directly into a CED 1401 and filtered with a 100 Hz fourth order lowpass Butterworth filter, and with a 50‐Hz notch filter, and the position signal from the Biodex was first passed through a Digitimer NeuroLog NL 125 Bandpass filter prior to being passed into the CED 1401. This analog filtering of the position signal was necessary to clean the signal for setting an accurate position threshold for the dynamic (i.e., concentric and eccentric) muscle action conditions.

### 3.3 CMEP Amplitude

The peak‐to‐peak amplitude of the CMEP responses was calculated for the conditioned CMEP and the unconditioned CMEP. The CMEPs were then expressed as a percentage of the muscle action specific *M*_max_ [CMEP (%*M*_max_)]. Additionally, the mean of the 10 conditioned CMEPs was calculated as a percentage of the mean of the 10 unconditioned CMEP within a muscle action type to calculate RST excitability.

### 3.4 Prestimulation Background EMG

The root mean square (RMS) of the EMG signal was calculated for a 100‐ms window immediately preceding each electrical stimulation to assess background muscle activity.

## 4 Statistical Analyses

Repeated measures analysis of variance (RM‐ANOVA) tests were conducted for data that met assumptions, and a Friedman's test was conducted for data that violated data assumptions. The first test was a single factor three level (muscle action; isometric, concentric, eccentric) RM‐ANOVA to assess the difference in stimulation intensity. The second was a 3 (muscle action; isometric, concentric, eccentric) × 2 (stimulus; unconditioned CMEP, conditioned CMEP) RM‐ANOVA with the primary outcome variable being the peak‐to‐peak CMEP responses recorded at the *biceps brachii* expressed as a percentage of *M*_max_. The third Friedman's test assessed RST excitability as a single factor with three levels of muscle action type (isometric, concentric, eccentric) with the primary outcome being changes in CMEP amplitude from an auditory stimulus (Conditioned CMEP as a percentage of unconditioned CMEP). Finally, pre‐stimulation background EMG was also assessed with a 3 (muscle action; isometric, concentric, eccentric) × 2 (stimulus; unconditioned CMEP, conditioned CMEP) RM‐ANOVA to determine if muscle activity prior to stimulation differed between muscle action types. The alpha level for significance testing was set to *α* = 0.05. Partial eta squared ($\eta_{p}^{2}$) effect sizes were reported for ANOVA tests (effect size convention: small ≤ 0.06; medium ≥ 0.07 and ≤ 0.13; large ≥ 0.14 (Cohen 1988)), and Kendall's *W* effect sizes were reported for Friedman's test. Mauchly's test of Sphericity and Shapiro–Wilk's test of normality were used to assess data sphericity and normality, respectively. Statistical analyses were performed in JASP v.0.19.1 (Team J 2024).

## 5 Results

### 5.1 Data Assumptions

Mauchly's test of sphericity and Shapiro–Wilk's test of normality were conducted for the three separate analyses. The assumption of sphericity was not violated for any analysis; however, data normality was violated for the RST excitability analysis (isometric: *S‐W* = 0.566, *p* < 0.001; concentric: *S‐W* = 0.850, *p* = 0.022; eccentric: *S‐W* = 0.802, *p* = 0.005). Therefore, RM‐ANOVA tests were conducted for the stimulation intensity and the muscle action type × stimulus CMEP (%*M*_max_) interaction, and a nonparametric Friedman's test was conducted for the RST excitability analysis.

### 5.2 Stimulation Intensity

A RM‐ANOVA to assess the CMEP stimulation intensity (mA) between isometric (140.39 ± 40.68 mA), concentric (141.39 ± 42.93 mA), and eccentric (147.85 ± 44.46 mA) muscle actions did not reach significance (*F*_2,24_ = 2.637, *p* = 0.092, $\eta_{p}^{2}$ = 0.180).

### 5.3 CMEP (%*M*_max_) Interaction

**FIGURE 3:**
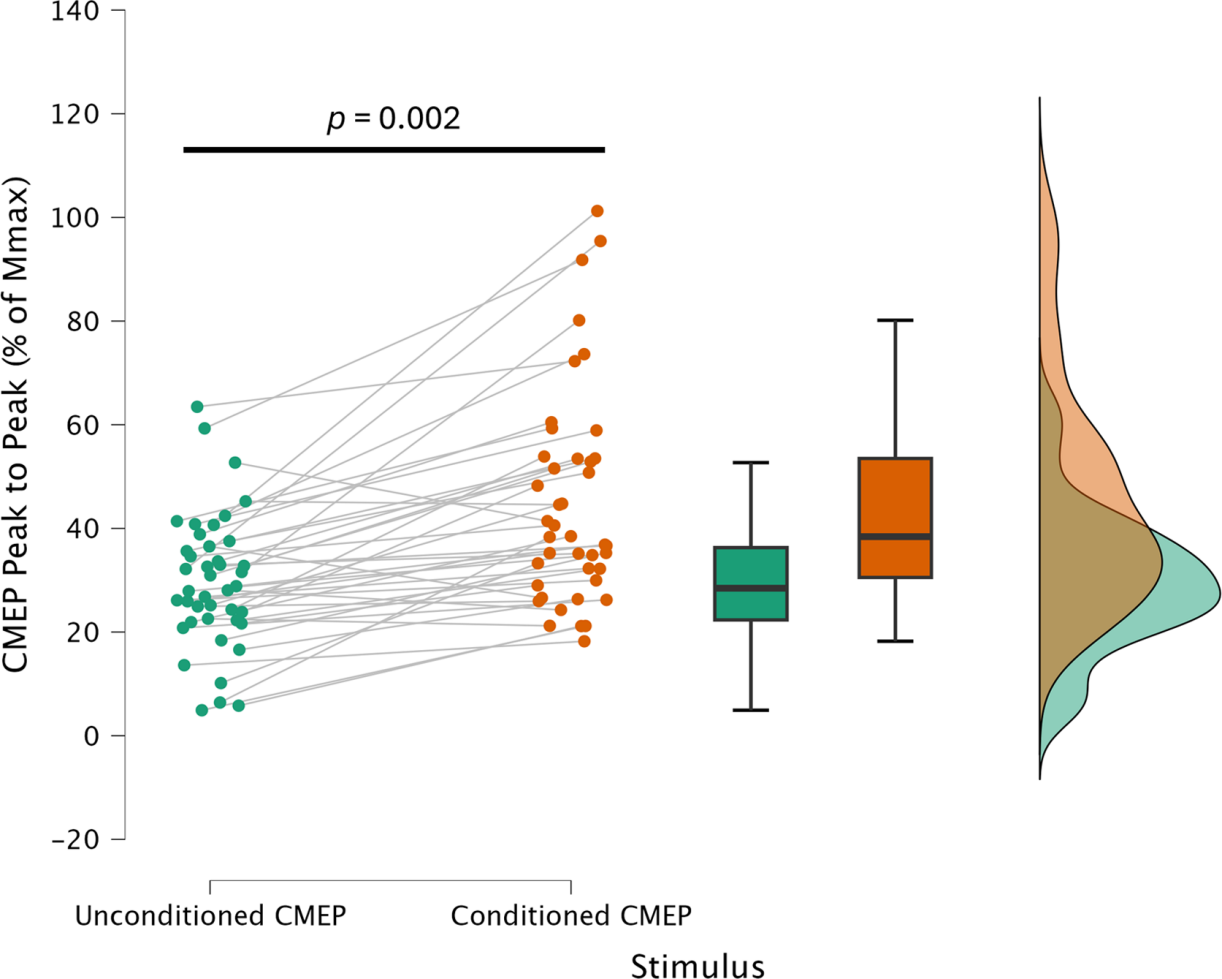
Unconditioned (i.e., no loud sound) cervicomedullary motor evoked potentials (CMEP) expressed as a percentage of *M*_max_ are represented by the green circles (left), with conditioned CMEP responses shown by the orange circles (right). The box plots represent the median CMEP peak‐to‐peak (% of *M*_max_) response for each condition, and the edges of the box represent the interquartile range of the data from 25th (bottom edge of box) to 75th percentile (top edge of box). The violin plot on the far right represents the distribution of the data for each stimulation condition. Main effect of stimulus is reported by the black horizontal bar, *p* = 0.008, $\eta_{p}^{2}$ = 0.432.

The 3 (muscle action; isometric, concentric, eccentric) × 2 (stimulus; unconditioned CMEP, conditioned CMEP) RM‐ANOVA, interaction (*F*_2,26_ = 0.009, *p* = 0.991, $\eta_{p}^{2}$ < 0.001), and main effect of muscle action (*F*_2,26_ = 0.286, *p* = 0.754, $\eta_{p}^{2}$ = 0.022) were not significant. However, a significant main effect of stimulus was observed (*F*_1,26_ = 13.948, *p* = 0.008, $\eta_{p}^{2}$ = 0.432), which demonstrated a significant difference between conditioned CMEP and unconditioned CMEP stimuli (Figure 3). An additional simple main effects analysis revealed significant differences between conditioned CMEP and unconditioned CMEP for all muscle action types (Table 1, far right column).

**TABLE 1:** Stimulation peak‐to‐peak responses.

Muscle action	Stimulus	N	*M*_max_ mV	CMEP % *M*_max_	95% CI	SE	CMEP % *M*_max_ simple main effects
Isometric	Unconditioned	14	3.979 ± 3.241	29.617 ± 9.094	24.366–34.868	2.430	*F*_1_ = 15.988, *p* = 0.002
	Conditioned	14		44.906 ± 17.069	35.051–54.761	4.562	
Concentric	Unconditioned	14	4.216 ± 3.298	27.976 ± 11.696	21.223–34.729	3.126	*F*_1_ = 10.790, *p* = 0.006
	Conditioned	14		43.516 ± 18.502	32.834–54.199	4.945	
Eccentric	Unconditioned	14	4.426 ± 3.309	31.178 ± 17.181	21.258–41.098	4.592	*F*_1_ = 11.527, *p* = 0.005
	Conditioned	14		46.389 ± 26.678	30.985–61.792	7.130	

*Note:* Data in table are presented as mean ± standard deviation. *M*_max_ values were not significantly different between muscle actions (*p* = 0.164, $\eta_{p}^{2}$ = 0.137).

### 5.4 RST Excitability

**FIGURE 4:**
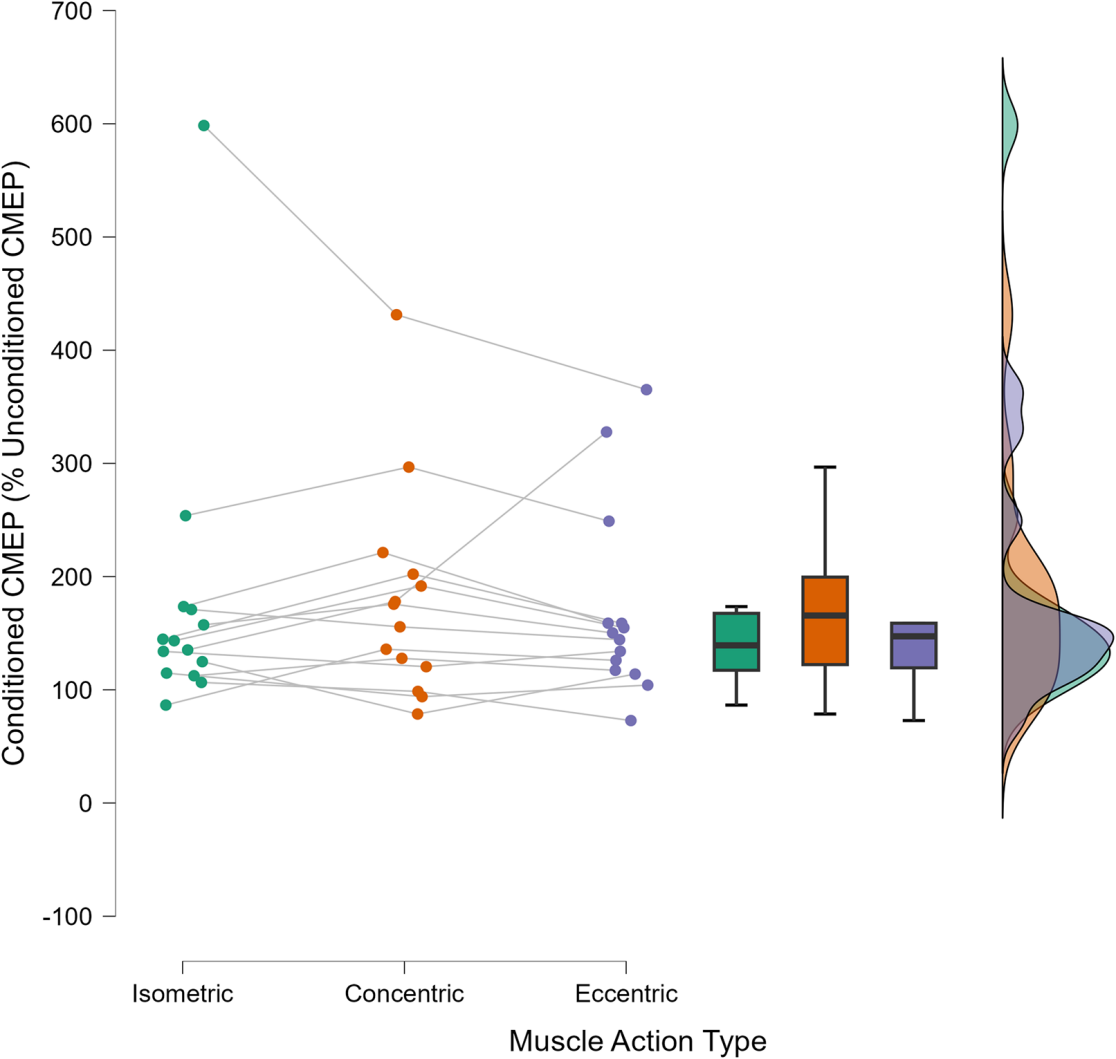
RST excitability is represented as conditioned cervicomedullary motor evoked potentials (CMEP) and expressed as a percentage increase from the unconditioned CMEP. Isometric muscle actions are represented by the green dots, concentric actions by the orange dots, and eccentric muscle actions are represented by the purple dots. The box plot and violin plot represent the mean and distributions of the data for each condition. There were no significant differences between muscle action type.

**FIGURE 5:**
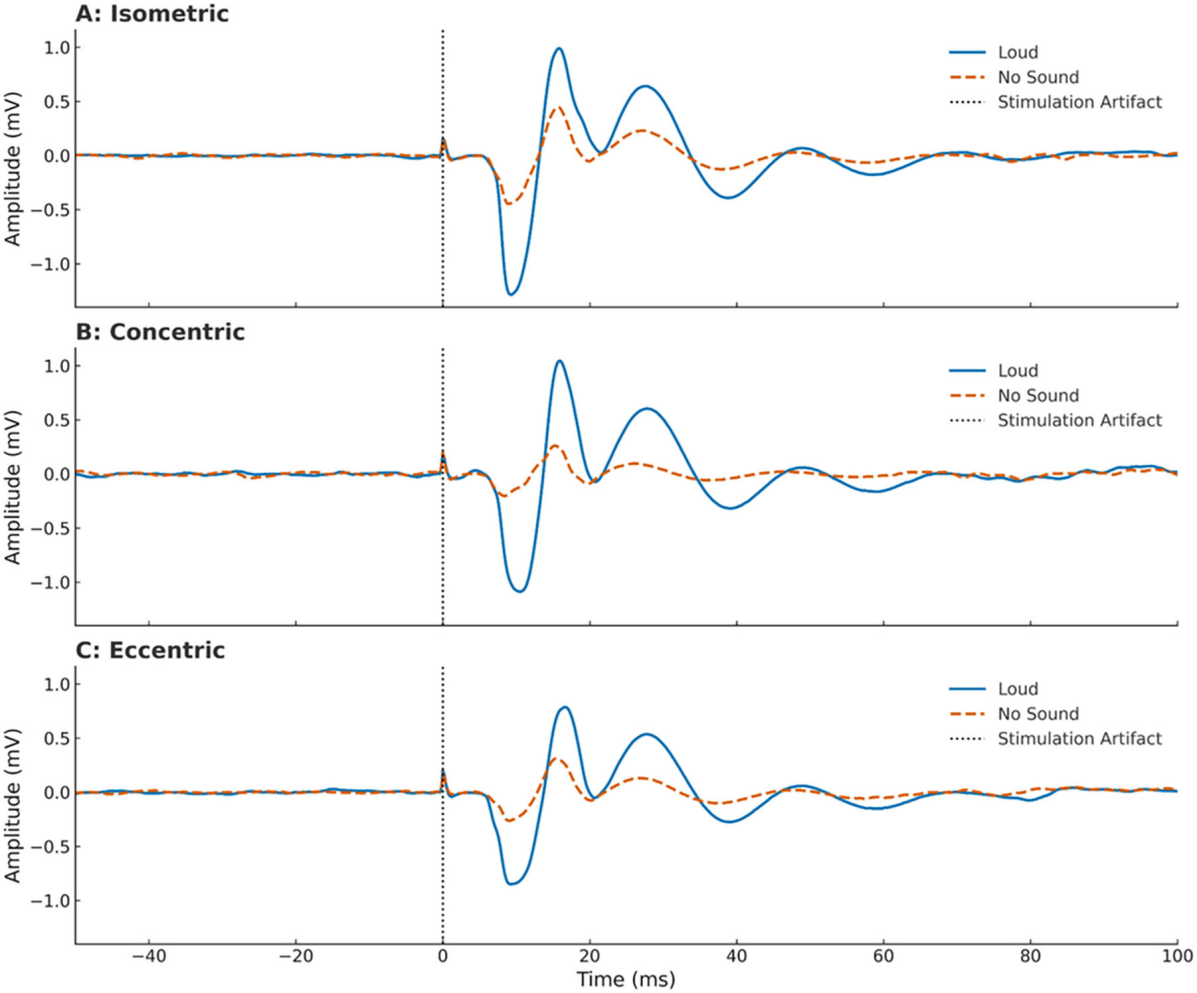
Cervicomedullary motor evoked potentials (CMEP) responses for (A) isometric, (B) concentric, and (C) eccentric muscle actions during conditioned loud (blue lines), and unconditioned no sound (orange dashed lines) conditions.

The single factor, three level muscle action type (isometric, concentric, eccentric) CMEP amplitude Friedman's test did not detect differences between muscle action type (*X* ^2^(2) = 2.286, *p* = 0.319, *W* = 0.082; Figures 4 and 5).

### 5.5 Presimulation Background EMG

A RM‐ANOVA revealed no significant main effect of muscle action on RMS EMG values, with isometric (mean ± SE; 0.043 ± 0.011 mV), concentric (0.050 ± 0.011 mV), and eccentric (0.047 ± 0.012 mV) muscle actions showing no significant differences (*F*_2,26_ = 2.665, *p* = 0.089, $\eta_{p}^{2}$ = 0.170). A significant main effect of stimulus condition was observed (*F*_1,13_ = 16.169, *p* = 0.001, $\eta_{p}^{2}$ = 0.554) with RMS EMG values being higher for conditioned CMEPs (0.052 ± 0.013) compared with unconditioned CMEP (0.041 ± 0.010). However, the interaction between muscle action and stimulus condition was nonsignificant (*F*_2,26_ = 0.131, *p* = 0.878, $\eta_{p}^{2}$ = 0.010).

## 6 Discussion

The present study is the first to investigate RST excitability to the biceps brachii during submaximal isometric, concentric, and eccentric elbow flexion movements. Using the conditioned CMEP approach, we were able to indirectly measure RST excitability across all three muscle actions, as evidenced by the significant differentiation between conditioned and unconditioned CMEP responses. Although RST excitability was able to be assessed across the three muscle action types, the findings of this study reveal no difference in the RST excitability to the *biceps brachii* during isometric, concentric, and eccentric muscle actions during the low intensity actions at 25% of isometric MVC.

We hypothesized that RST excitability would differ between muscle action types, with eccentric actions producing the lowest RST excitability due to previous research demonstrating a reduction in cortical output compared with concentric and isometric actions (Doguet et al. 2017), and a reduction in motoneuron excitability due to muscle spindle afferents indirectly inhibiting cortical output (and thus potentially impacting descending motor commands via the cortico‐reticulospinal tract onto the reticular formation) (Kably and Drew 1998; Keizer and Kuypers 1989; Kuypers 1958), and also increasing pre‐synaptic inhibition (Gruber et al. 2009; Skarabot et al. 2019a, 2019b; Wolstencroft 1964; Del Valle and Thomas 2005), which modulates both spinal reflex loops and subcortical circuits, including the RST, through ascending indirect spinal interneuronal mechanisms (Wolstencroft 1964).

Previous research has found that motor unit discharge rates change depending on the muscle action type (Del Valle and Thomas 2005; Pasquet et al. 2006). Studies utilizing intramuscular EMG of the *tibialis anterior* (Pasquet et al. 2006) and *triceps brachii* (Del Valle and Thomas 2005) observed differing motor discharge rates during concentric and eccentric actions, with lower discharge rates during eccentric actions, and comparable discharge rates between concentric and isometric muscle actions (Del Valle and Thomas 2005). Del Valle and Thomas (2005) suggested this reduction in motor unit discharge rates during eccentric actions may be the result of some protective mechanism to prevent muscle damage, presumably by presynaptic Ia afferent inhibition from the muscle spindles, reducing the excitability of alpha motoneurons. These observations are congruent with research findings examining spinal excitability, where studies have also found smaller Hoffmann (H)‐reflex responses during eccentric actions (Abbruzzese et al. 1994; Nordlund et al. 2002). Additionally, a recent study by Ruas et al. (2024) observed lower voluntary activation during eccentric, compared with concentric and isometric muscle actions during a knee extension task. Ruas et al. (2024) also investigated muscle action type differences during lower force actions at 30% of MVC. During low‐force muscle actions at 30% of isometric MVC, the corticospinal silent period was shorter during eccentric contractions; however, no differences in corticospinal excitability were observed. Collectively, these observations are indicative that the neuromuscular properties of muscle contractions differ depending on the type of muscle action being performed. However, the lack of differences in RST excitability between muscle action types in the present study may indicate the RST does not contribute differently to different muscle action types at the same absolute force during low force efforts.

In the present study, by maintaining the same absolute force (25% of isometric MVC) across isometric, concentric, and eccentric muscle actions, we inherently introduced variability in the relative contraction intensity between these actions, where concentric and eccentric actions would be performed at a higher and lower relative action type specific intensity, respectively (Skarabot et al. 2018). The observed lack of RST modulation across muscle actions with consistent absolute force output might offer insight into the RST's role in force generation. It is plausible that the RST provides a generalized, nonspecific neural drive that is subsequently refined by the CST. This concept aligns with the hypothesis put forward by Glover and Baker (Glover and Baker 2022), who recorded neural activity in the reticular formation of two macaque monkeys, finding that RST activity correlated with general force output. In contrast, recordings of corticospinal cells appeared to contribute to more specific, fine‐scale adjustments to the force output (Glover and Baker 2022). Consequently, in the present study, given that participants maintained the same absolute force across all muscle actions, the RST neural drive might not necessitate differences. With this hypothesis, any required modulation in force output between actions would likely come from alternative pathways such as the CST, where distinct differences in corticospinal excitability have been observed (Skarabot et al. 2019a; Duclay et al. 2014).

## 7 Limitations and Future Directions

In this study, we elected to maintain a fixed relative force output across the three muscle action types, by setting the target for all three muscle actions to 25% of each participant's isometric MVC. Whist this approach allowed us to investigate if RST excitability differed between muscle actions at the same absolute force, this decision introduced a limitation in that the concentric and eccentric muscle actions were performed at a higher and lower relative intensity respectively (Skarabot et al. 2018), and future research should consider investigating RST excitability differences between muscle actions at the same relative intensity. Additionally, having the participants perform the respective contractions at the relatively low 25% of isometric MVC may have “masked” any true differences given the RST is most excitable during higher intensities (Danielson et al. 2024; Glover and Baker 2022), and the lack of differences in the present study cannot necessarily be generalized to higher contraction intensities. Future studies might consider attempting to replicate this work during higher intensity efforts, however based on our piloting for this study, differentiating CMEP responses from background EMG was difficult to detect during dynamic muscle actions at higher intensities. In the present study, for our supramaximal stimulations to evoke *M*_max_, we elected to deliver these stimulations at *M*_max_ + 10%, however it is important to note that higher stimulations are generally recommended with the current consensus being to deliver these stimulations at *M*_max_ + 20% (Osborne et al. 2023). Although we are confident our stimulations were maximal as was evident by the lack in increase in peak‐to‐peak amplitude at with the additional of the 10% current intensity, higher intensity stimulations would be preferred to ensure any changes in recruitment thresholds do not impact M‐wave measurements. Another important consideration for future research is to consider implementing a true randomization approach for the order of muscle actions being performed. In the present study, we implemented a quasi‐randomized approach, where we ensured eccentric actions were always performed last. We did this to minimize the potential impact of fatigue and muscle damage from subsequent eccentric actions. However, given the low intensity focus of this work (i.e., 25% of isometric MVC), the likelihood of fatigue or muscle damage occurring was minimal and future work might consider true randomization. Finally, given the nature of assessing RST function in humans requires the use of indirect methods, it is possible that the observed changes in excitability have other cortical or subcortical contributing factors, and alternative assessment approaches such as ipsilateral motor evoked potentials (Atkinson et al. 2022), or ipsilateral—contralateral amplitude ratios (ICAR) (Akalu et al. 2024; Maitland and Baker 2021; Hu et al. 2024), might produce different results, and should be considered in future work.

## 8 Conclusion

This study is the first to investigate the potential differences in RST excitability across concentric, isometric, and eccentric muscle actions of the *biceps brachii* during elbow flexion movements. Here, using conditioned CMEP responses, we demonstrate that RST excitability can be measured across different muscle actions during a 25% of isometric MVC. However, there were no differences in RST excitability between muscle actions, despite our hypothesis that there would be lower RST excitability during eccentric actions. These findings indirectly support the findings by Glover and Baker (Glover and Baker 2022) where the RST appears to provide a generalized neural output for force production, where using the same absolute force across the three muscle action types did not necessitate differences in RST output.

## Author Contributions

EH, EA, and JWA collected the research data. EA and JWA prepared the first draft of the manuscript. EA and JWA pre‐processed the data. JWA carried out the statistical analyses and prepared figures. EA, PA, RV, KT, SG, GH, LA, DJK, PS, ES, and JWA codesigned the study, reviewed, and approved the manuscript for publication.

## Conflicts of Interest

The authors declare no conflicts of interest.

## Peer Review

The peer review history for this article is available at https://www.webofscience.com/api/gateway/wos/peer‐review/10.1111/ejn.70205.

## Supporting information


**Data S1.** Supporting Information

## Data Availability

Data will be made available for all reasonable requests.
