# Supplementary material for: There Are No Differences in Startle Conditioned Cervicomedullary Motor Evoked Potentials Across Isometric, Concentric, and Eccentric Muscle Actions at the Same Absolute Force Output
Source: Eur J Neurosci. 2025 Aug 4;62(3):e70205. doi: 10.1111/ejn.70205 (PMC12319894; doi:10.1111/ejn.70205)
Supplement: Supplementary file 1 — Data S1. Supporting Information [file EJN-62-0-s001.docx]

**Additional statistical analysis of background EMG.**

Given that the sound from the auditory stimuli was within the 100 ms window prior to stimulation, of which the EMGrms was calculated, an additional analysis of the background EMGrms was calculated using a 25 ms window prior to stimulation, which avoids the potential for contamination of the auditory tone in the signal.

This analysis produced consistent results with the 100 ms pre stimulation window. There was a non-significant main effect of muscle action type [*F*(1,12.95) = 3.176, *p* = 0.075], however the main effect of condition (sound vs no sound) remained significant [*F*(1, 12.99) = 9.951, *p* = 0.008).

With this finding, it appears the sound does cause an increase in EMG activity as well as an increase in electrically evoked responses. It is possible the increase in EMG activity is reflective of the startle reflex that is thought to be mediated by the reticulospinal tract.

| *Estimated Marginal Means* of the 25 ms window prior to stimulation | | | | | | | | | | | |
| --- | --- | --- | --- | --- | --- | --- | --- | --- | --- | --- | --- |
|  | | | | | | | | 95% CI | | | |
| Contraction | | Condition | | Estimate | | SE | | Lower | | Upper | |
| Concentric |  | Loud |  | 0.077 |  | 0.020 |  | 0.038 |  | 0.116 |  |
| Eccentric |  | Loud |  | 0.067 |  | 0.019 |  | 0.030 |  | 0.105 |  |
| Isometric |  | Loud |  | 0.062 |  | 0.016 |  | 0.031 |  | 0.094 |  |
| Concentric |  | No Sound |  | 0.049 |  | 0.012 |  | 0.025 |  | 0.073 |  |
| Eccentric |  | No Sound |  | 0.040 |  | 0.011 |  | 0.018 |  | 0.061 |  |
| Isometric |  | No Sound |  | 0.034 |  | 0.008 |  | 0.019 |  | 0.050 |  |

**Isometric CMEP onset latency analysis**

To determine whether spinal root activation occurred during the stimulation intensities used for data collection, CMEP onset latencies were compared across three isometric conditions: (1) across the lower intensities used to identify CMEP thresholds ("CMEP Intensity Search"), during stimulation sufficient to evoke CMEPs approximately 30% of Mmax paired with a loud auditory stimulus ("CMEP Loud"), and the same stimulation level delivered without sound ("CMEP No Sound"). A shift in latency would indicate premature spinal root activation. However, a repeated measures ANOVA revealed no differences between CMEP latency, [*F*_1.044, 13.566_ = 0.378, *p* = 0.558, $\eta_{p}^{2}$ = 0.028]. Mean onset latencies were comparable across conditions: 9.45 ± 0.76 ms (CMEP Intensity Search), 9.74 ± 1.95 ms (CMEP Loud), and 9.34 ± 0.85 ms (CMEP No Sound). These findings suggest that the stimulation intensity used for data collection did not induce spinal root activation, as no systematic latency shift was observed.

| *Descriptives* | | | | | | | | | | | |
| --- | --- | --- | --- | --- | --- | --- | --- | --- | --- | --- | --- |
| CMEP | | N | | Mean | | SD | | SE | | Coefficient of variation | |
| CMEP Intensity Search |  | 14 |  | 9.454 |  | 0.756 |  | 0.202 |  | 0.080 |  |
| CMEP Loud |  | 14 |  | 9.744 |  | 1.946 |  | 0.520 |  | 0.200 |  |
| CMEP_No_Sound |  | 14 |  | 9.344 |  | 0.848 |  | 0.227 |  | 0.091 |  |
